# A seamless living biointerface for inflammation management

**DOI:** 10.1093/nsr/nwae268

**Published:** 2024-08-05

**Authors:** Junyi Yin, Shaolei Wang, Guorui Chen, Xiao Xiao, Jun Chen

**Affiliations:** Department of Bioengineering, University of California, Los Angeles, USA; Department of Bioengineering, University of California, Los Angeles, USA; Department of Bioengineering, University of California, Los Angeles, USA; Department of Bioengineering, University of California, Los Angeles, USA; Department of Bioengineering, University of California, Los Angeles, USA

## Abstract

A novel living biointerface that integrates living biological and hydrogel systems, can significantly improve monitoring and treatment through enhanced interaction with biological tissues, revolutionizing our chronic inflammation management.

The biointerfaces of traditional electronic devices are typically composed of rigid metals or semiconductors, which significantly differ from human body tissues in various aspects. The mechanical, electrical, and physiological disparities between biological tissues and electronic devices often restrict their applications in treating and monitoring complex diseases [1]. Furthermore, the intricate and dynamic nature of biological tissues and cells poses significant challenges in achieving the necessary cellular functions for tissue regulation using traditional materials [2]. Consequently, current bioelectronics lack the bio-originated capability to modulate immune responses while monitoring disease states, limiting their multifunctionality in addressing the complexities of various diseases [3].

Living biological systems, such as bacteria and mammalian cells, naturally possess the ability to generate and transmit cellular signals [4]. Recent technologies have bridged the gap between electronic devices and living biological systems. For example, networks of genetically programmed bacteria integrated with hydrogel structures have been developed to receive and process signals (Fig. 1a) [5]. By incorporation with multiple chemosensory cells, these living materials can adhere to human skin to monitor various chemical substances by emitting green fluorescence in designated patterns when they are exposed to these chemicals (Fig. 1b). Additionally, these living sensors are suitable for oral use, facilitating long-term diagnostics of intestinal diseases [6] (Fig. 1c). The active component, genetically modified *Escherichia coli*, expresses bioluminescence when encountering extracellular hemin in the surrounding medium (Fig. 1d). Encased in magnetic hydrogels, these sensors are directed to specific intestinal areas using magnetic guidance, ensuring continuous monitoring. The chemical permeability of the hydrogel matrix supports bacterial growth and survival, allowing for the detection of gastrointestinal bleeding through hemin sensing in the harsh intestinal environment.

**Figure 1.**
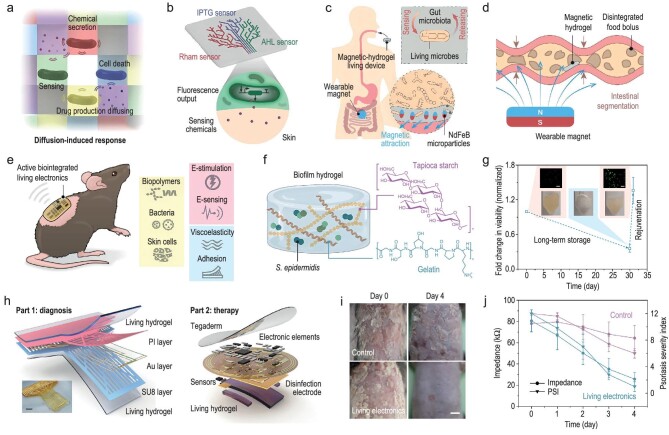
Active biointegrated living bioelectronics for inflammation management. (a) Illustration of live bacteria reacting to chemical diffusion within the hydrogel substrate. (b) Living materials adhering to skin surfaces for chemical detection. Reprinted with permission from Wiley-VCH [5]. (c) Ingestible magnetic living sensors for precise localization and monitoring within the gastrointestinal tract. (d) Magnetic hydrogel living sensors maintain structural integrity during intestinal peristalsis. Reprinted with permission from Wiley-VCH [6]. (e) Biointegrated living electronics interact among bioelectronics, hydrogels, and bacteria. (f) Construction of a dual-network hydrogel living biointerface using protein and polysaccharide polymers. (g) Long-term bacterial preservation in the hydrogel matrix with overnight revival. Scale bar, 20 μm. (h) Configuration of the bioelectronic system based on the living hydrogel interface. Scale bar, 5 mm. (i) Images of living electronic treatment for psoriasis on Day 0 and Day 4. Scale bar, 5 mm. (j) Impedance measurements of skin lesions using living electronics indicate psoriasis recovery. Reprinted with permission from the American Association for the Advancement of Science [7].

The living biointerfaces serve as the bridge for signal transmission and regulation between biological and electronic components, enabling multimodal signal transduction at the microorganism-tissue interface (Fig. 1e). These living biointerfaces are composed of diverse layers, including an electronic layer and a hydrogel composite layer containing live bacteria [7]. Skin commensal bacterium (*S. epidermidis*) can be utilized as living components of the biointerface, endowing bioelectronics with the ability to modulate inflammation and promote skin regeneration. To support the long-term survival of *S. epidermidis*, the hydrogel matrix uses a dual network composed of protein (gelatin) and polysaccharide polymers (tapioca starch) (Fig. 1f). The hydrogel supports bacterial growth, and after rehydration and overnight incubation at room temperature, the bacteria's viability, which was lost during storage, can be restored and even exceed initial levels (Fig. 1g). Additionally, the living hydrogel exhibits excellent bioelectrical and biomechanical properties, allowing for seamless integration with tissues due to its tissue-level mechanical characteristics. The mechanical and electrochemical properties of the living hydrogel also remain stable during long-term electrophysiological recordings.

Psoriasis is a chronic inflammatory disease affecting ∼125 million people worldwide. It remains incurable and current treatments are facing challenges of high costs and numerous side effects [8]. Electronics with living biointerface have recently been demonstrated in mouse models with psoriasis to evaluate their physiological signal recording and disease treatment functions. First, a mesh-like electronic device with a living interface provides (Part 1) qualitative monitoring of psoriasis symptoms by recording the surface electrocardiograms (sECG) (Fig. 1h). Meanwhile, a wireless flexible printed circuit board (FPCB, Part 2), integrated with radiofrequency energy harvesting, wireless data transmission, and sensing functions, monitors psoriasis in real-time and treats it with living biological components (Fig. 1i). The FPCB monitors disease recovery progress by recording impedance, humidity, and temperature of the skin (Fig. 1j). It also provides information for bacterial regulation, allowing for sterilization management via an electrode-delivered current to prevent infection and mitigate the potential long-term impacts of commensal bacterial proliferation and colonization on skin health.

Living bioelectronic technology offers a novel approach for the future treatment of various diseases. It provides a new method to establish the interactions between biological and non-biological systems. In the future, living bioelectronics is expected to merge with synthetic biology, exploring how devices incorporating engineered microorganisms can release inflammation-resolving factors and support therapeutic fine-tuning. This technology progresses towards adaptive medicine by regulating the living environment of bacteria and facilitating controlled interactions with the host. Moreover, the integration of living cells with therapeutic functions and bioelectronics for inflammation monitoring is a key advancement towards living bioelectronics with closed-loop functionality [9], capable of balancing microbial therapeutic effects and adverse reactions through the emulation of innate homeostatic regulation. Further research into the molecular mechanisms underlying disease can offer a broad application of living bioelectronics beyond psoriasis to other inflammatory conditions, providing an adaptable paradigm for the control of inflammation and related epidemic diseases [10].

However, living biointerfaces also face several practical challenges in the transitional stage: (1) *Limited wireless functionality*: while current technologies facilitate wireless energy harvesting and data transmission, these capabilities are predominantly confined to the treatment end (Part 2). The wireless performance at the diagnostic end (Part 1) remains suboptimal and could benefit from further advancements in integrated circuit and chip designs to enable fully wireless operations [11]. (2) *Power consumption and thermal management*: increased power consumption may generate heat that affects the surrounding tissues, raising bio-safety concerns. Developing low-power designs or efficient thermal management technologies is crucial to ensure biocompatibility and safety. (3) *Breathability concerns for skin diseases*: prolonged use of living bioelectronic devices may affect skin breathability, potentially causing skin issues, especially for individuals with sensitive skin. These challenges can be mitigated by using breathable device designs, such as textile structures or sweat-wicking channels [12–14]. (4) *Motion artifacts in living bioelectronics*: when using ECG to monitor psoriasis symptoms qualitatively, it is inevitable to capture biopotential motion artifacts such as electromyography. Advanced signal processing strategies, like filtering and machine learning, can be employed to enhance the elimination of artifact signals [15]. Addressing these challenges is crucial for enhancing the effectiveness and practicality of living bioelectronic devices in clinical settings.
